# Death of Dr. I. P. Lynn, Etc.

**Published:** 1866-03

**Authors:** Thos. Bevan

**Affiliations:** Sec’y.


DEATH OF DR. I. P. LYNN.—At a meeting of the profession, held at the Council Chamber, Chicago, March 2d, 1866, on the occasion of the death of Dr. I. P. Lynn, Dr. R. C. Hamill was elected Chairman, and Dr. Thos. Bevan, Secretary.

On motion, Drs. Allen, Blake and Miller were appointed a committee to draft suitable resolutions in relation to the melancholy event.

Dr. E. Ingalls made a brief address, eulogistic of the estimable qualities of our departed confrere.

Dr. J. A. Allen, from the Committee on Resolutions, reported as follows:

*Resolved*, That the intelligence of the sudden and untimely death of our late friend and co-worker in the medical profession,

Isaiah P. Lynn, M. D., is received by this meeting with emotions of profound pain and sorrow.

*Resolved*, That the memory of our deceased brother will be cherished by us as of one who, personally, and by his amiable disposition and fine friendly feeling, endeared himself to all who knew him intimately; whilst, by his conscientious earnestness in acquainting himself with the principles of medical science, and thorough devotedness to the welfare of those who entrusted themselves to his care as patients, he commanded the confidence, the respect and esteem of both the profession and the public.

*Resolved*, That we tender to his bereaved family and friends, the assurances of our warmest sympathy in this their great affliction.

*Resolved*, That copies of these resolutions be presented to the relatives, the public press and medical journals of this city for publication.

On motion, the report was accepted and adopted unanimously.

On motion of Dr. Hay, it was resolved that the profession attend the funeral in a body.

Dr. J. A. Allen then occupied the meeting with appropriate remarks, highly creditable to the amiable character, kindness and faithfulness of the lamented dead. He also referred to the peculiarly sad circumstances of the immediate cause of death, giving an entirely satisfactory explanation of the accident which had resulted so unfavorably.

Dr. V. L. Hurlbut followed Dr. Allen in a similar vein.

Drs. Brooks and McVickar referred feelingly to the courtesy and worth of the deceased, Dr. M. giving some incidents of his personal relations to the Doctor, of a very pleasant character.

Drs. N. S. Davis, S. Wickersham and D. L. Miller, also addressed the meeting, expressing their cordial endorsement of the resolutions, and of the sentiments of sympathy and respect which had been expressed by other speakers.

On motion, the meeting adjourned.

THOS. BEVAN, M. D., *Sec'y.*

---

MARRIED.—On February 13th, 1866, at the residence of the bride's parents, Moline, Ill., by Rev. A. B. Hitchcock, assisted by Rev. A. H. Lackey, Dr. R. M. LACKEY, one of the Editors of this Journal, and Miss FRANK A. HITCHCOCK.

